# A systematic review of the knowledge, attitude and practice of healthcare professionals and healthcare professional students towards household pharmaceutical waste disposal

**DOI:** 10.1016/j.rcsop.2024.100556

**Published:** 2024-12-19

**Authors:** Sheng Yuan Hiew, Bee Yean Low

**Affiliations:** School of Pharmacy, University of Nottingham Malaysia, Jalan Broga, 43500 Semenyih, Selangor, Malaysia.

**Keywords:** Knowledge, Attitude, Practice, Healthcare professionals, Household pharmaceutical waste, Disposal

## Abstract

**Background:**

Active pharmaceutical residues introduced into the environment through irresponsible household disposal of unused and expired medication can damage nature and health. Through medication take-back programmes, these risks can be mitigated. However, healthcare professionals' neglect of their responsibility to ensure proper disposal practices has perpetuated subpar norms among the public.

**Objectives:**

The objectives of this systematic review were to assess the knowledge, attitude, and practice of household pharmaceutical waste disposal among healthcare professionals and healthcare professional students as well as to compare their respective levels of knowledge, attitude and practice.

**Methods:**

A systematic search of published articles from 2014 to 2023 in three online journal databases (Pubmed, Scopus, and Web of Science) yielded an initial pool of 10,381 records, which was narrowed down by title and abstract screening to 46 relevant publications for full-text examination and the final inclusion of 21 papers for data extraction and synthesis.

**Results:**

The findings revealed deficiencies in academic curricula and medication disposal training, leading to inadequate knowledge and perceptions among healthcare professionals. Many healthcare professionals consider public education about safe medication disposal practices to be outside their job descriptions, leading to infrequent communication of medication disposal methods to their consumers and service users. Additionally, inadequate and inaccessible infrastructure further hinders proper medication disposal practices despite awareness of the consequences.

**Conclusions:**

This study provides insights for policymakers and educators to address these and enhance healthcare professionals' participation in improving safe medication disposal practices. Efforts to strengthen training programmes, incorporate comprehensive education on medication disposal into curricula, and improve infrastructure for safe medication disposal are essential to effectively address the issue of household pharmaceutical waste disposal.

## Highlights

• Healthcare professionals lack awareness of proper disposal channels.
• Under half of the professionals received formal safe disposal education.
• Professionals seldom teach patients despite recognising responsibility.
• Pharmacists advocate for community pharmacy collection depots.
• Healthcare professional students have poor disposal habits.

## 1 Introduction

Household pharmaceutical waste (HPW) refers to unused and expired medication (UEM) commonly found at home. This UEM results from various factors such as non-adherence to medication regimens, discontinuation due to improved health, and oversupply from medical sources.^1^^,^^2^ Proper disposal of this unwanted medication is vital due to its prolonged pharmacological activity even after its expiry, posing environmental risks. Studies in the Baltic Sea region have highlighted improper disposal of household medication as a major contributor to pharmaceutical pollutants in wastewater, as contemporary treatment facilities struggle to eliminate such residues effectively.^3^ The primary methods of inappropriate HPW disposal globally include disposal in household garbage, toilets and sinks.^4^^,^^5^ Flushing is an exception recommended by the Food and Drug Administration (FDA) for opioid medications in the absence of a take-back programme due to their high abuse potential. The FDA considers the environmental risks associated with the flush list medications to be negligible, while also emphasizing the environmental unsustainability of this practice and urging that other medications should not be flushed.^6^ To prevent and reduce the release of active pharmaceutical ingredients (APIs) into the environment, numerous countries have implemented medication take-back programmes, offering secure drop-boxes, mail-back services, and designated collection points in drugstores or pharmacies for community members to return HPW safely.^7^, ^8^, 9., 10, 11 The World Health Organization has issued guidelines for the safe disposal of unwanted pharmaceuticals, particularly in and after emergencies.^12^ The guideline suggested that solid and semi-solid pharmaceutical wastes can be treated by incineration, encapsulation or landfilling at engineered sites, while liquid pharmaceutical wastes with no or low toxicity can be diluted and flushed into a sewer. A review by Sapkota and Pariatamby indicated that incineration is a sustainable option for solid and semi-solid pharmaceutical wastes, while wastewater treatment plants are considered eco-friendly for liquid pharmaceuticals; however, encapsulation and landfilling are deemed less sustainable.^13^ In certain countries, legislative frameworks have been introduced to reduce environmental pollution through structured collection systems for unwanted pharmaceuticals. For instance, Directive 2004/27/EC of the European Parliament mandates all EU members to implement a form of collection system for expired medicinal products.^14^ Similarly, Brazilian Decree N. 10.388/2020 regulates the reverse logistics of expired or unused medicines from Brazilian homes^15^ and the Australian Environmental Protection Regulation 2019 designates pharmaceutical waste as regulated waste to be destroyed through high-heat incineration.^16^

Healthcare professionals, particularly pharmacists are commonly regarded as reliable sources of information on medication disposal for the public.^17^ Pharmacists can contribute greatly to preventing API escape into the environment by ensuring compliance and adherence to reduce medication wastage, and by providing convenient and accessible disposal facilities for their local communities. Aside from pharmacists, doctors and nurses are also in positions to closely advise patients on returning their unused and unwanted medications. However, survey studies involving pharmacists and members of the public worldwide have revealed common findings that healthcare providers often fail to provide adequate instructions and counselling to patients regarding the proper management of their unused and expired medications.^18^, ^19^, ^20^, ^21^, ^22^, ^23^, ^24^ This prominent lack of proactive involvement among healthcare professionals in ensuring appropriate disposal practices among their patients has likely contributed to poor awareness among the public.^25^

According to the knowledge, attitude, and practice (KAP) model, knowledge nurtures the development of attitudes, which then influence practice.^26^ Healthcare professionals, with their expertise in drug utilisation and management, are expected to possess extensive knowledge regarding the environmental and health impacts of improper HPW disposal, along with a nuanced understanding of disposal techniques for unused and expired medications. Ideally, this knowledge should foster positive attitudes towards responsible HPW management, leading to proactive personal disposal practices, such as returning medication at higher rates than the general public and advocating for proper disposal methods. Similarly, university-level students, particularly those in healthcare disciplines like medicine, pharmacy, and nursing, are expected to have a solid foundation in KAP regarding HPW disposal, which they can further develop and apply in their future roles as healthcare professionals. This collective commitment to sound HPW management among current and future healthcare practitioners underscores their role in educating and promoting responsible disposal practices within their communities. However, despite the importance of this issue, there remains a notable gap in research assessing the KAP of healthcare professionals and healthcare professional students regarding HPW disposal, highlighting the need for comprehensive studies to elucidate how personal attitudes and practices influence their efforts to encourage safe disposal practices among the public.

Given the above, this systematic review aims to address the research questions: What are the levels of KAP of healthcare professionals and healthcare professional students concerning HPW disposal? Is there any significant difference in KAP levels among categories of healthcare professionals and healthcare professional students across various courses of study? Are the KAP levels regarding HPW disposal among healthcare professionals greater than among healthcare professional students studying health sciences? How do personal KAP levels and training received in school affect practices and involvement in patient education? An understanding of the current position of practising and upcoming healthcare professionals can be attained by critically analysing research articles assessing the levels of KAP among the above population. The insights from this review are instrumental in devising appropriate interventions and strategies to remedy the deficiencies surrounding healthcare professional involvement in guiding the public towards safe medication disposal practices.

### 1.1 Objectives

The objectives of this systematic review were to assess the knowledge, attitude, and practice (KAP) of HPW disposal among healthcare professionals and healthcare professional students as well as to compare their respective levels of KAP.

## 2 Methods

### 2.1 Study design

This systematic review adhered to the Preferred Reporting Items for Systematic Reviews and Meta-Analyses (PRISMA) guidelines. The PRISMA Checklist for this systematic review is available in Supplementary Material 1. The protocol registration number for this review in the PROSPERO International Prospective Register of Systematic Reviews is CRD42024535559.

### 2.2 Search strategy

The databases used for this review are PubMed, Scopus, and Web of Science. The keyword combination used for the search terms is available in Supplementary Material 2. The search was limited to research articles published in English from January 2014 to mid-November 2023.

### 2.3 Inclusion and exclusion criteria

This review specifically incorporated research articles reporting cross-sectional, quantitative survey studies that examined at least one of the three components of KAP related to HPW disposal among healthcare professionals and university students from healthcare and non-healthcare fields. Mixed-method studies were included only if there was distinctive reporting of quantitative results separately from the qualitative findings. Publications focusing on the general public or the disposal of biomedical waste (including sharps, infectious, and hazardous waste) were excluded from this review.

### 2.4 Selection process

Duplicates in the search results were removed through Microsoft EndNote using the duplicate filter. Subsequently, all the search results were screened through titles and abstracts, completed independently by both authors to select potentially relevant citations. The screened results were then compared and harmonised by discussion, followed by the retrieval of full-text online and an in-depth review against the inclusion criteria. Disagreements regarding the selection of articles were resolved through a structured process that included the following steps: (1) clarification of the inclusion and exclusion criteria, focusing on the article's scope and methodology; (2) discussion of the rationale for each article's inclusion or exclusion based on these criteria; (3) thorough examination of the evidence presented in the articles against the inclusion and exclusion criteria; and (4) documentation of the reasons for each article's final status, as the authors reached a consensus.

### 2.5 Risk of bias assessment

The articles selected for final inclusion in this review were assessed for methodological quality and the possibility of bias using the Appraisal Tool for Cross-Sectional Studies (AXIS).^27^ The AXIS tool comprises a 20-item checklist developed for appraising observational cross-sectional studies. The risk of bias assessment of the included articles in this review was conducted independently by both authors, with any discrepancies resolved through reconciliation and mutual agreement.

### 2.6 Data extraction

The extraction of the data was performed independently by both authors and any inconsistent opinions were discussed and settled via consensus. The following relevant data was extracted from the articles included in this review:

1. Authors and year of study publication
2. Study population and its subgroups
3. Geographical location of the study
4. Sampling method and sample size
5. Study design
6. Research instrument source and instrument validation
7. Outcome measures of individual knowledge, attitude and practice

Given the diversity in KAP assessment methods among the articles, the outcome measures for each component were determined based on the most pertinent and frequently used questions identified within the articles included in this review. The specific questions or aspects evaluated for each KAP component are outlined below. The reported percentages or data for the specific question or aspect assessed were extracted from the articles included in this review.

Knowledge

(i) Knowledge of medication disposal methods.
(ii) Awareness towards the negative impact on the environment and health.
(iii) Received training or advice regarding safe medication disposal.

Attitude

(i) Attitude towards health care professionals' responsibility in ensuring safe disposal.
(ii) Attitude towards medication take-back programmes in pharmacies (healthcare professionals only)

Practice

(i) Personal medication disposal practices (healthcare professional students only)
(ii) Professional management of medication disposal at the workplace including patient returns (healthcare professionals only)
(iii) Advising others on safe medication disposal or accepting medication returns from patients (healthcare professionals only)

## 3 Results

The search across online databases produced a total of 10,381 records, which were subsequently exported to Endnote. Following this, 1128 duplicate records were removed, along with 4 retracted articles. The remaining 9249 records underwent screening based on their titles and abstracts for relevance. After excluding 9203 irrelevant records, the remaining 46 potentially relevant articles were then acquired in full text and evaluated against the inclusion and exclusion criteria. Finally, 21 eligible cross-sectional, survey-based research articles were selected for data extraction. These comprised 11 articles from Pubmed, 2 from Scopus, and 8 from Web of Science. The elimination process for this review is depicted in a PRISMA flow diagram, available in Supplementary Material 3.

The predominant demographic subgroups found in studies focused on healthcare professionals and healthcare professional students were pharmacists and pharmacy students, respectively. Out of the 21 studies reviewed, eleven specifically involved healthcare professionals, including pharmacists, nurses, doctors, or various combinations thereof.^19^^,^^25^^,^^28^, ^29^, ^30^, ^31^, ^32^, ^33^, ^34^, ^35^, ^36^ Nine studies concentrated solely on university students enrolled in either general or healthcare professional courses.^37^, ^38^, ^39^, ^40^, ^41^, ^42^, ^43^, ^44^, ^45^ Additionally, one study in India in the year 2018 reported combined responses from both healthcare professionals (doctors and nurses) and healthcare professional students (medical and pharmacy courses).^46^ Geographically, this review covered principal regions such as Africa,^34^^,^^35^^,^^37^^,^^41^ Middle East,^29^^,^^30^^,^^33^^,^^38^^,^^40^ South Asia,^28^^,^^31^^,^^36^^,^^42^^,^^43^^,^^46^ Southeast Asia,^25^ Caribbean,^19^ Europe,^32^^,^^44^ and the USA,^39^^,^^45^ providing a diverse representation of healthcare professionals and healthcare professional students on a global scale.

The characteristics of the studies included in this review and their extracted KAP outcomes are organised and presented in Table 1, Table 3 for healthcare professional students, Table 2, Table 4 for healthcare professionals respectively.

**Table 1:** Characteristics of healthcare professional student respondents.

First author, Year	Study population	Subgroups	Geographical location	Sampling method, sample size	Sample size	Study design	Research instrument source	Instrument validation
Akande-Sholabi et al.,^37^ 2023	Healthcare professional students	Medical and surgery, nursing, pharmacy, physiotherapy, and medical laboratory science students	Nigeria	Convenience sampling	*N* = 930	Cross-sectional, self-administered online questionnaire	Questionnaire derived from Ayele & Mamu^59^	Pre-tested and validated
Alhomoud et al.,^38^ 2021	Pharmacy undergraduate or postgraduate Students	Pharmacy undergraduate or postgraduate students	Saudi Arabia	Convenience and snowball sampling	*N* = 464	Descriptive, cross-sectional, online questionnaire.	Questionnaire adopted from Owens & Anand^60^	Pre-validated and pre-tested
Al Rawwad et al.,^39^ 2021	Professional pharmacy (Pharm.D.) and nursing students	Professional pharmacy (Pharm.D.) and nursing students	Houston, USA.	Purposive sampling,	*N* = 210 [139 Pharm.D., 71 nursing students]	Exploratory, descriptive, and cross-sectional online questionnaire	Self-designed questionnaire adapted from Tong et al.,^61^ Pankajkumar et al. and Bataduwaarachchi et al.^62^	Validated and pilot-tested
Bashatah and Wajid,^40^ 2020	Senior-level (4th, 5th and 6th year) pharmacy and nursing students at King Saud University	Pharmacy and nursing students	Riyadh, Saudi Arabia.	Convenience sampling,	*N* = 352 [161 pharmacy, 191 nursing students]	Cross-sectional, self-administered paper questionnaire	Self-designed questionnaire based on Lucca et al.,^63^ and Raja et al.^46^	Validated and pilot-tested
Gubae et al.,^41^ 2023	Third to fifth year pharmacy students from Debre Tabor University (DTU), University of Gondar (UoG), Bahir Dar University (BDU), and Debre Markos University (DMU).	Pharmacy students	Northwestern Ethiopia	Convenience sampling	*N* = 445	Descriptive, cross-sectional, self-administered questionnaire.	Self-designed questionnaire adapted from Liu et al.,^64^ Bhatt and Kumar,^65^ and Advani and Jadhao^66^	Pre-validated and pre-tested.
Jha et al.,^42^ 2021	Undergraduate students in KIST Medical College and Teaching Hospital	Medical (MBBS) and dental (BDS) students	Lalitpur, Nepal.	Convenience sampling	*N* = 441	Cross-sectional, self-administered online questionnaire	Self-designed questionnaire based on Paudel et al.,^67^ Helal and Abou-Elwafa,^68^ Angi'enda and Bukachi^69^	Validated
Raja et al.,^46^ 2018	Medical and pharmacy students (and doctors and staff nurses) at SRM Medical College Hospital & Research Centre	Medical and pharmacy students (and doctors and staff nurses)	Chennai, Tamil Nadu, India	Convenience sampling	*N* = 393	Cross-sectional, questionnaire	Self-designed questionnaire	Not reported
Shakib et al.,^43^ 2022	Students studying in the top 10 private universities in Bangladesh	Pharmacy, Engineering, and Business Administration	Bangladesh	Convenience sampling	*N* = 250 [150 pharmacy, 100 general students]	Descriptive, cross-sectional, conducted through face-to-face interviews.	Self-designed, questionnaire.	Validated
Shuleta-Qehaja and Kelmendi,^44^ 2022	Undergraduate students from a medical college	Pharmacy and nursing students	Kosovo, Southeast Europe	Random sampling	*N* = 336, [169 nursing, 167 pharmacy students]	Descriptive, cross-sectional, self-administered questionnaire	Self-designed questionnaire adapted from Raja et al.,^46^ Braund et al.,^70^ Tong et al.,^71^ Glassmeyer et al.,^7^ Persson et al.,^72^ Kusturica et al.,^73^ Vellinga et al.,^18^ Azad et al.,^74^ Bashatah and Wajid^40^	Pre-tested and validated.
Vatovec et al.,^45^ 2017	University students at the University of Vermont	Not specified	Vermont, USA	Convenience sampling	*N* = 358	Cross-sectional online questionnaire	Self-designed questionnaire	Survey tool previously published in Vatovec et al.,^75^ survey validation not reported

**Table 2:** Characteristics of healthcare professional respondents.

Author, Year	Study population	Subgroups	Geographical location	Sampling method, sample size	Sample size	Study design	Research instrument source	Instrument validation
Aditya and Rattan,^28^ 2014	Pharmacists working in pharmacies in an urban town	Pharmacists	North India	Simple random sampling	*N* = 84	Descriptive, cross-sectional, structured questionnaire.	Self-designed questionnaire	Pre-tested
Albaroodi,^29^ 2019	Pharmacists in the government sector (Al-Hussain General Hospital; Gyn./Obstetrics Hospital) and pharmacists working in private pharmacies at Karbala	Public sector (hospital) and Private sector pharmacists	Karbala, Iraq.	Convenience sampling	*N* = 129	Cross-sectional, self-administered questionnaire	Self-designed questionnaire based on WHO,^76^ Abahussain et al.,^77^ Aditya and Rattan, 2014,^28^ WHO, 2004, Salkin and Kennedy,^78^ Manojlovic et al.,^79^ Lamb,^80^ Shukla et al.^81^	Pre-tested and validated
Alghadeer and Al-Arifi,^30^ 2021	Community pharmacist in Riyadh city	Community pharmacists	Saudi Arabia	Convenience sampling	*N* = 360	Cross-sectional, self-administered questionnaire.	Self-designed questionnaire adopted from Tong et al.,^61^ and Abahussain et al.^77^	Pilot tested to assess validity and reliability.
Bhayana et al.,^31^ 2016	Medical doctors, nurses and pharmacists working at Lady Hardinge Medical College and associated tertiary care public hospitals. Qualified pharmacists working at private pharmacies in the nearby vicinity of the hospitals.	Medical doctors, nurses, and pharmacists	New Delhi, India	Convenience sampling	*N* = 300	Cross-sectional, mixed methods questionnaire, instructor-administered	Self-designed questionnaire	Pre-tested
Bungau et al.,^32^ 2018	Pharmacists practising across the country	Pharmacists	Romania	550 pharmacists sampled from 17,850 pharmacists	*N* = 521	Cross-sectional questionnaire by phone or online.	Self-designed questionnaire.	Pilot tested
Jankie et al.,^19^ 2022	Public and private pharmacists employed in Trinidad	Public sector and private sector pharmacists	Trinidad	Randomised interval sampling	*N* = 208	Cross-sectional, self-administered electronic questionnaire	Self-designed questionnaire adapted from Tong et al.,^61^ drug disposal guidelines of the WHO^76^and United States Food and Drug Administration (USFDA).^6^	Pilot tested
Kharaba et al.,^33^ 2022	Licensed community pharmacists working in the UAE for two or more years	Community pharmacists	United Arab Emirates (UAE)	Convenience sampling	*N* = 418	Cross-sectional questionnaire	Questionnaire adopted from Tong et al.^61^	Pilot tested and validated
Low et al.,^25^ 2023	Pharmacists attending the Malaysian Community Pharmacy Guild (MCPG) event in person	Community pharmacists	Malaysia	Convenience sampling	*N* = 168	Cross-sectional, self-administered questionnaire	Self-designed questionnaire	Pre-tested
Mahlaba et al.,^34^ 2021	Healthcare professionals from 16 randomly selected primary health-care clinics (PHCs) as representatives from 30 clinics in the region.	Nurses and other healthcare professionals	City of Tshwane, Gauteng Province	Convenience sampling	*N* = 166	Descriptive, cross-sectional, quantitative, self-administered questionnaire.	Self-designed questionnaire based on Glassmeyer et al.,^82^ Abruquah et al.,^83^ Tong et al.,^61^ and Seehusen and Edwards^50^	Pilot tested
Michael et al.,^35^ 2019	All registered community pharmacists	Community pharmacists	Anambra state, southwest Nigeria	No sampling used (census)	*N* = 77	Self-administered quantitative questionnaire	Questionnaire adapted from Tong et al.^61^	Pre-tested and revalidated
Raja et al.,^46^ 2018	Doctors, staff nurses (and medical and pharmacy students) at SRM Medical College Hospital & Research Centre	Doctors and staff nurses (and medical and pharmacy students)	Chennai, Tamil Nadu, India	Convenience sampling	N = 393	Cross-sectional, questionnaire	Self-designed questionnaire	Not reported
Sarraf et al.,^36^ 2022	Healthcare professionals working at B.P. Koirala Institute of Health Sciences (BPKIHS)	Faculties and junior resident	Dharan, Nepal	Random sampling	*N* = 294	Descriptive, cross-sectional, semi-structured online questionnaire.	Self-designed questionnaire in accordance with the relevant literature (Jha et al.,^42^ Bhayana et al.,^31^ Ayele and Mamu,^59^ Gupta et al.,^84^ Michael et al.^35^	Pre-tested and validated

**Table 3:** Knowledge, attitude and practice outcomes of healthcare professional student respondents.

Author, Year	Student Subgroup	K1: Knowledge of medication disposal methods	K2: Awareness towards the negative impact on the environment and health	K3: Received training or advice regarding safe medication disposal	A1: Attitude towards healthcare professionals' responsibility in ensuring safe disposal	P1: Personal medication disposal practices⁎
Akande-Sholabi et al.,^37^ 2023	Mixed healthcare professional students	60.1 % aware that returning medicines to pharmacy or drug take-back programme is the safest disposal method	Contamination of water supplies and aquatic ecosystems (83 %), accumulation of pharmaceutical residues in soil (74.3 %), development of antibiotic-resistant bacteria (62.3 %), increased risk of accidental ingestion (83.5 %)	16.2 % had received training on safe disposal (83.8 % had not received)	Not assessed	10.1 % correct, 89.9 % incorrect
Alhomoud et al.,^38^ 2021	Pharmacy undergraduate or postgraduate students	48.5 % aware that prescription and non-prescription medications should be disposed of differently (knowledge of disposal methods not reported)	87.5 % agree improper disposal could impact the environment and human health	40 % received information (60 % had never) on storage and disposal during studies/training	81.5 % believe pharmacists are responsible for safe disposal, 69.5 % believe doctors and other healthcare providers are responsible, FDA (90 %) and pharmaceutical companies (87 %)	15 % correct, 85 % incorrect
Al Rawwad et al.,^39^ 2021	Pharm.D. students	Aware of medication's bin collected by contractors (75.5–82.0 %)	Not assessed	53 % received advice (47 % did not) on proper disposal of medications	Not assessed	Correct: 38.3 % (liquid), 39.2 % (tablet/ capsules), 25.2 % (ointment/creams) Incorrect: 61.7 % (liquid), 60.8 % (tablet/ capsules), 74.8 % (ointment/creams)
	Nursing	Aware of medication's bin collected by contractors (73.0–85.9 %)	Not assessed	31.1 % received advice (68.9 % did not) on proper disposal of medications	Not assessed	Correct: 21.7 % (liquid), 28.3 % (tablet/ capsules), 15 % (ointment/creams) Incorrect: 78.3 % (liquid), 71.7 % (tablet/ capsules), 85 % (ointment/creams)
Bashatah and Wajid,^40^ 2020	Pharmacy students	Not assessed	81.8 % aware improper disposal can affect the environment and health	Not assessed	19.9 % believe pharmacists are responsible for creating awareness, 64.6 % chose Ministry of Health	Unused medicines: 6.8 % correct, 93.2 % incorrect Expired medicines: 8.1 % correct, 91.9 % incorrect
	Nursing students	Not assessed	91.9 % aware improper disposal can affect the environment and health	Not assessed	16.3 % believe pharmacists are responsible for creating awareness, 81.6 % chose Ministry of Health	Unused medicines: 4.7 % correct, 95.3 % incorrect Expired medicines: 2.6 % correct, 97.4 % incorrect
Gubae et al.,^41^ 2023	Pharmacy students	Not assessed	Aware about environmental pollution (59.8 %), endangering ecosystem and wildlife (54.4 %), antimicrobial resistance (42.2 %)	27.0 % had been taught in pharmacy school or received information from healthcare professionals	90.1 % believe pharmacists are responsible for protecting the environment from pharmaceutical waste	6.1 % correct, 93.1 % incorrect
Jha et al.,^42^ 2021	Medical students	Median score 8/10 covering knowledge about medicine disposal, takeback system and hazards caused by improper disposal of medicines, etc.		Not assessed	Not assessed	Unused medicines: 9.3 % correct, 90.7 % incorrect
	Dental students	Median score 9/10 covering knowledge about medicine disposal, takeback system and hazards caused by improper disposal of medicines, etc.		Not assessed		
Raja et al.,^46^ 2018	Medical and Pharmacy students	Data is represented collectively with healthcare professionals in Table 4.				
Shakib et al.,^43^ 2022	Mixed pharmacy and general (engineering and business administration) students	36.4 % heard of standard drug disposal methods (46 % of pharmacy students, 21 % of general students)	62.8 % know about the environmental hazards because of the improper disposal of medicines (72.7 % of pharmacy students, 48 % of other students)	Not assessed	78 % believe pharmacists are required for counselling on proper disposal, 19.4 % perceive pharmacists as the best source of awareness (percentages quoted representing mixed pharmacy and general students)	14 % correct, 86 % incorrect (liquid) 11.2 % correct, 88.8 % incorrect (solid) (percentages quoted representing pharmacy and general students) Following required standard disposal method: 8 % solid, 13.3 % liquid (pharmacy students), 0 % solid, 5 % liquid (general students)
Shuleta-Qehaja and Kelmendi,^44^ 2022	Pharmacy students	Not assessed	95.2 % aware inappropriate disposal affects health and the environment	Not assessed	29.9 % believe pharmacists are responsible for disposal, 64.1 % chose Ministry of Health	Unused medicines: 11.4 % correct, 88.6 % incorrect Expired medications: 14.4 % correct, 85.6 % incorrect
	Nursing students	Not assessed	97.0 % aware inappropriate disposal affects health and the environment	Not assessed	35.5 % believe pharmacists are responsible for disposal, 67.5 % chose Ministry of Health	Unused medicines: 10.7 % correct, 89.3 % incorrect Expired medications: 10.1 % correct, 89.9 % incorrect
Vatovec et al.,^45^ 2017	Mixed general students	24 % had heard of National Drug Take-Back Day and 4 % had utilized the service	Not assessed	Not assessed	Not assessed	2 % correct, 26 % incorrect

⁎ Correct disposal refers to returning medicines to pharmacies, incineration and/or specific medication disposal products/bags, incorrect disposal refers to all other disposal methods such as discarding into trash, toilets, sinks, giving away, burning, keeping until expired, etc.

**Table 4:** Knowledge, attitude and practice outcomes of healthcare professional respondents.

Author, Year	Subgroup	K1: Knowledge of medication disposal methods	K2: Awareness towards the negative impact on the environment and health	K3: Received training or advice regarding safe medication disposal	A1: Attitude towards healthcare professionals' responsibility in ensuring safe disposal	A2: Attitude towards medication take-back programmes in pharmacies	P2: Professional management of medication disposal at the workplace including patient returns	P3: Advising others on safe medication disposal or accepting medication returns from patients
Aditya and Rattan,^28^ 2014	Pharmacists	33 % aware garbage disposal for solid and semi-solid drugs, and sinks (55 %) and flushing (56 %) for liquids are not acceptable. 69 % deduced incineration was environmentally sound.	58 % aware improper drug disposal affects the environment and ecosystem	11 % were taught proper drug disposal techniques in pharmacy school (89 % not taught)	Not assessed	Not assessed	Return to distributor: 69 % solids, 84 % semi-solids, 86 % liquids, 94 % controlled drugs, 93 % P-listed drugs Garbage: 29 % solids, 15 % semi-solids, 5 % liquids, 1 % controlled drugs, 4 % P-listed drugs Incineration: 2 % controlled drugs, 2 % P-listed drugs Sink: 5 % liquids Others: 2 % solids, 5 % liquids, 2 % controlled drugs, 1 % P-listed drugs	Not assessed
Albaroodi,^29^ 2019	Pharmacist	65.9 % aware drugs should be returned to source, 58.9 % aware of take-back programme	Not assessed	Not assessed	Not assessed		Not assessed	
Alghadeer and Al-Arifi,^30^ 2021	Community pharmacists	Not reported	78.9 % aware that drug disposal damages the environment	Not reported	87.5 % acknowledged personal responsibility of community pharmacists	Not reported	Return to distributor: 75.3 % solid, 73.3 % liquid, 74.2 % semi-solid Medicines bin: 16.1 % solid, 15.5 % liquid, 16.7 % semi-solid Rubbish: 3.6 % solid, 1.7 % liquid, 3.3 % semi-solid	Not reported
Bhayana et al.,^31^ 2016	Doctor	59 % know about disposal methods	Not assessed	Not assessed	95 % believe everyone^a^ is responsible	Not assessed	Not assessed	8.0 % of HCPs received unused/expired medications back from consumers.
	Nurse	76 % know about disposal methods			94 % everyone^a^ is responsible			
	Pharmacist	70 % know about disposal methods			66 % everyone^a^ is responsible			
Bungau et al.,^32^ 2018	Pharmacists	Not assessed			Not assessed	65 % dissatisfied with the current procedure. 62.57 % suggested special containers in pharmacies for direct disposal by citizens	Not assessed	32.82 % have cases of refusing medicine collection from public
Jankie et al.,^19^ 2022	Private sector pharmacist (community)	42.7 % think OTC medicines can be disposed of in household trash	76.2 % aware of soil and water contamination, 44.1 % knew about improper disposal of antibiotics can cause bacterial resistance	Not assessed	23.8 % believe pharmacists are responsible for providing drug disposal information	Not assessed	83.9 % return to pharmaceutical distributor, 60.8 % incinerate through drug inspectorate, 37.8 % dispose in workplace garbage	20.3 % give advice on medicine disposal
	Public sector pharmacist	40 % think OTC medicines can be disposed of in household trash	86.2 % aware of soil and water contamination, 47.7 % knew about improper disposal of antibiotics can cause bacterial resistance	Not assessed	38.5 % believe pharmacists are responsible for providing drug disposal information		73.8 % return to pharmaceutical distributor, 70.8 % incinerate through drug inspectorate, 20 % dispose in workplace garbage	72.3 % give advice on medicine disposal
Kharaba et al.,^33^ 2022	Community pharmacists <10 years exp	Aware of correct disposal⁎ methods used by contractors (33.3 %) and distributors (31.5 %)	Not assessed	Not assessed	Not assessed	68.4 % believe the United Arab Emirates (UAE) needs specialised centre for medication disposal.	Return to distributor: 29 % solid, 33.3 % semi-solid, 34.5 % liquid Contractors: 45.3 % solid, 40.1 % semi-solid, 41.2 % liquid Rubbish bin: 15.4 % solid, 9.6 % semi-solid, 7.9 % liquid	Not assessed
	Community pharmacists >10 years exp.	Aware of correct disposal⁎ methods by contractors (28.6 %) and distributors (33.7 %)	Not assessed	Not assessed	Not assessed		Return to distributor: 23.1 % solid, 17.6 % semi-solid, 19.6 % liquid Contractor: 32.2 % solid, 33.6 % semi-solid, 36.7 % liquid Rubbish bin: 14.1 % solid,13.7 % semi-solid, 10.2 % liquid	Not assessed
Low et al.,^25^ 2023	Community Pharmacist	85.7 % aware landfill disposal is unsafe, 80.4 % know disposal into household waste is unsafe	42.9 % aware aquatic organisms are more susceptible to pharmaceuticals than humans	56.5 % received education (43.5 % never received)	86.9 % believe community pharmacists are responsible to ensure correct disposal of patient medication	94.1 % believe a Medication Return Programme should be set up nationwide	Not assessed	10.7 % have promotional materials encouraging safe medication disposal in their pharmacy
Mahlaba et al.,^34^ 2021	Mixed HCPs	12.8 % knowledgeable regarding correct disposal method	Not assessed	28.9 % received training (10.8 % formally, 18.1 % informally)	Not assessed		Not assessed	27.7 % always and 42.8 % sometimes counsel patients regarding safe disposal of medicines
Michael et al.,^35^ 2019	Community Pharmacists	Not assessed			Not assessed		Return to wholesaler/distributor: 31.8 % solid, 34.1 % liquid, 33.0 % semi-solid, 24.7 % Class B controlled drug, 30.1 % Class C controlled drug NAFDAC bin: 35.2 % solid, 31.8 % liquid, 33.0 % semi-solid, 29.6 % Class B controlled drug, 36.1 % Class C controlled drug Incorrect: 33 % solid, 34.2 % liquid, 34.2 % semi-solid, 45.6 % Class B controlled drug, 33.7 % Class C controlled drug	Not assessed
Raja et al.,^46^ 2018	Doctors, staff nurses (and medical and pharmacy students)	23 % aware of the national take-back programme abroad (77 % not aware)	89 % aware that improper disposal causes harm	Not assessed	13 % believe doctors are responsible for creating awareness, 79.6 % believe doctors and the government should work together	Not assessed	Not assessed	(Healthcare professionals) 26 % always advise patients, 43 % advise patients occasionally, 31 % never advise patients
Sarraf et al.,^36^ 2022	Faculties and junior residents	53.7 % aware that returning medicines to hospital, pharmacies or manufacturer is the best recommended method for safe disposal method	94.2 % agree improper disposal of unused, unwanted and expired medicines adversely affects the environment and health	Not assessed	Not assessed	93.5 % agree a drug take-back system is required	Not assessed	Not assessed

⁎ Correct disposal referring to returning medicines to pharmacies, incineration, and/or specific medication disposal products/bags, incorrect disposal referring to all other disposal methods such as discarding into trash, toilets, sinks, giving away, burning, keeping until expired, etc.

a Definition of ‘everyone’ is not specified in the article.

**Table 5:**
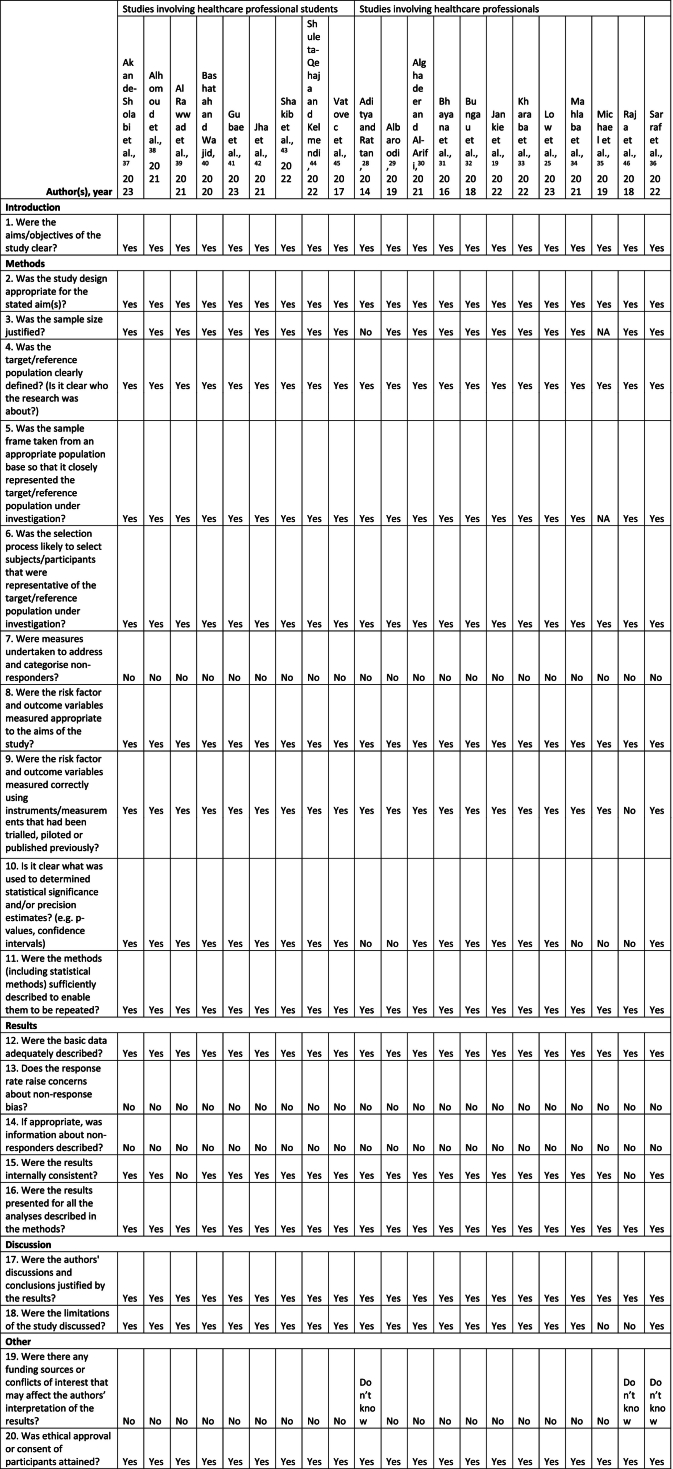
Risk of bias assessment using Appraisal tool for Cross-Sectional Studies (AXIS) for articles included in this review.

### 3.1 Knowledge, attitude and practice of healthcare professional students

#### 3.1.1 Healthcare professional students' knowledge of drug disposal methods

Healthcare professional student respondents' knowledge of correct drug disposal methods ranged from 46.0 % to 85.9 %.^37^, ^38^, ^39^^,^^42^^,^^43^ Pharmacy student respondents from the United States of America (USA) were mostly aware (ranging from 75 % to 82 %) that a medication bin by contractors was the correct way to dispose of various drug formulations. This awareness was similarly observed among nursing students in the same study (ranging from 73 % to 86 %).^39^ Over 60 % of mixed healthcare professional student respondents (from various healthcare courses e.g. pharmacy, nursing, medicine, etc) in a Nigerian study knew that returning medicines was the best channel for disposal.^37^ However, only a quarter of general (non-healthcare specific) student respondents in the USA were aware of local drug recollection programmes such as the National Drug Take Back Day.^45^ Furthermore, only 21 % of general (from engineering and business administration) students in Bangladesh had heard of standard drug disposal methods, compared to 46 % of pharmacy students who were aware of such methods.^43^

#### 3.1.2 Healthcare professional students' knowledge of environmental hazards

Seven studies identified an overall satisfactory level of awareness regarding the impact of improper disposal on the environment and health among healthcare professional students.^37^^,^^38^^,^^40^, ^41^, ^42^, ^43^, ^44^ Pharmacy students from Saudi Arabia and Southeast Europe particularly displayed a good understanding (over 80 %) that improper disposal incurred environmental damage,^38^^,^^40^^,^^44^ except for one study conducted in Northwestern Ethiopia that reported relatively weaker awareness about environmental pollution (59.8 %), the risk of harming wildlife (54.4 %) and bacterial resistance (42.2 %) among pharmacy students.^41^ Good environmental awareness was also revealed among other healthcare professional students (from nursing, medical, dental, etc.),^37^^,^^40^^,^^42^, ^43^, ^44^ and over 60 % of mixed healthcare professional students knew of detrimental effects such as aquatic pollution, accumulation in soil and bacterial resistance.^37^ However, the collective awareness level towards such environmental hazards was notably reduced to approximately 62 % when non-healthcare professional students were included as respondents along with pharmacy students (72.7 % of pharmacy students showed such awareness as opposed to 48 % of general students).^43^

#### 3.1.3 Disposal education and training received by healthcare professional students

The overall percentage of healthcare professional students who received training and advice regarding medication disposal was mostly low.^37^^,^^38^^,^^41^ Approximately 15 % to 16 % of mixed healthcare professional students in Nigeria (from various healthcare courses e.g. pharmacy, nursing, medicine, etc) had received such training,^37^ 27 % to 40 % of pharmacy students in Saudi Arabia and Northwestern Ethiopia were taught in pharmacy school,^38^^,^^41^ and 31 % of nursing students in an American study had ever received advice from healthcare professionals regarding safe disposal.^39^ Meanwhile, PharmD students in the USA were the only subgroup where slightly over half had received such drug disposal advice from healthcare professionals.^39^

#### 3.1.4 Healthcare professional students' attitudes towards the responsibility of healthcare professionals and safe disposal

Pharmacy students possessed mixed perceptions towards the responsibility of healthcare professionals in ensuring safe disposal as reported by four studies.^38^^,^^40^^,^^41^^,^^44^ The majority of pharmacy students (over 80 %) from studies conducted in Saudi Arabia and Northwestern Ethiopia believed that pharmacists are more responsible for handling HPW disposal^38^^,^^41^ compared to doctors and other healthcare professionals (69.5 %).^38^ Additionally, 78 % of mixed pharmacy and general students from a Bangladesh study believed that pharmacist counselling of patients regarding proper disposal was necessary.^43^ In contrast, one study conducted in Kosovo, Southeast Europe reported only 30 % of pharmacy students and 35 % of nursing students felt that pharmacists were responsible for safe disposal.^44^ Furthermore, approximately 20 % of pharmacy and nursing students from Riyadh, Saudia Arabia expressed sentiments that pharmacists were responsible for information dissemination to the public.^40^ Similarly, only 13 % of respondents (consisting of healthcare professionals and healthcare professional students) in a study in India held doctors accountable for spreading awareness towards safe disposal^46^ It should be noted that over 60 % of the pharmacy and nursing students in Riyadh and Kosovo believed that it was the role of the Ministry of Health instead to create awareness.^40^^,^^44^ while 80 % of the respondents from the study in Chennai, India called for the combined efforts of both the government and healthcare professionals.^46^ Overall, the attitudes towards safe drug disposal among pharmacy students can be considered positive, with 63.1 % having felt bothered by the thought of UEM disposal,^41^ 73 % proclaiming willingness to dispose of medicines in specific convenient locations,^38^ and 79.1 % agreeing that safe disposal places such as pharmacies are needed.^41^

#### 3.1.5 Healthcare professional students' personal disposal practices

Poor personal disposal practice was recorded among students regardless of their field of study, with approximately 10 % or less discarding their medications through correct channels. Pharmacy students' proper medication waste disposal practices (from 6 % to 15 %)^38^^,^^40^^,^^41^^,^^43^^,^^44^ were almost the same as medical and dental students (9 %),^42^ and nursing students (approximately 10 %).^44^ Only one study in the USA found relatively improved disposal practices among PharmD and nursing students (over 15 % for both); Up to 39 % of PharmD students practised responsible medicine waste management as opposed to the nursing students (under 28 %).^39^ However, the number of pharmacy students who followed the required standard disposal methods to eliminate their medicine waste (8 % to 13 %) was still greater than general students (less than 5 %) according to one study in Bangladesh.^43^ Additionally, 40.4 % of medical and dental students in a study in Nepal had educated their friends and family about safe medicine disposition,^42^ with as few as 15.5 % of mixed healthcare professional students (from various healthcare courses e.g. pharmacy, nursing, medicine, etc) in a study in Nigeria have advised others on safe disposal.^37^

### 3.2 Knowledge, attitude and practice of healthcare professionals

#### 3.2.1 Healthcare professionals' knowledge of disposal methods

A mixture of good and poor knowledge of disposal methods among healthcare professionals was found across eight studies.^19^^,^^25^^,^^28^^,^^29^^,^^31^^,^^33^^,^^34^^,^^36^ Pharmacists from a study in New Delhi, India (70 %) appeared to be knowledgeable about drug disposal methods,^31^ while pharmacists in Karbala, Iraq understood that unwanted drugs should be returned to their sources (65.9 %) or via a take-back programme (58.9 %).^29^ Malaysian pharmacists also knew that landfill disposal (85.7 %) and household waste (80.4 %) were unsafe disposal methods,^25^ and approximately 60 % of both private and public sector pharmacists from Trinidad recognised the unacceptability of disposing of OTC medications into trash.^19^ However, only 33 % of pharmacists in another study in North India were aware that garbage disposal for solid and semi-solid drugs was incorrect.^28^ In comparison, about half (53.7 %) of faculties and junior residents in Dharan, Nepal were aware that returning medications was the safest disposal method.^36^ Notably, doctors (59 %) had the weakest knowledge about drug disposal methods compared to nurses (76 %) and pharmacists (70 %).^31^ Furthermore, one study conducted in Africa found that only 12 % of miscellaneous healthcare professionals who participated in medication destruction knew about the correct disposal method of incineration,^34^ as opposed to 69 % of pharmacists correctly deducing that incineration was environmentally safe in a study conducted in North India.^28^ The study conducted by Aditya and Rattan in North India in the year 2014 found that 67 % of pharmacists believed that disposal of medications into garbage is acceptable, and just over half of them were aware that toilet and sink disposal of liquid medications was incorrect.^28^

#### 3.2.2 Healthcare professionals' knowledge of environmental hazards

Five studies reported a good level of environmental awareness among healthcare professionals.^19^^,^^25^^,^^28^^,^^30^^,^^36^ More than 58 % of pharmacists from North India and Saudi Arabia knew that improper disposal adversely affected the environment and health^28^^,^^30^ through soil and water contamination,^19^ with public-sector pharmacists exhibiting higher awareness (86.2 %) compared to private-sector community pharmacists in Trinidad (76.2 %).^19^ However, one study conducted in Malaysia in the year 2023 reported poor knowledge among community pharmacists with only 43 % of the respondents correctly answering that aquatic organisms were more susceptible to pharmaceuticals in water compared to humans.^25^ Among other healthcare professionals, both faculties and junior residents collectively reported a higher 94.2 % environmental awareness according to a study from Nepal,^36^ while 89 % of doctors and nurses (together with medical and pharmacy students) in a study in India recognised that improper pharmaceutical disposal could damage health and nature.^46^

#### 3.2.3 Disposal education and training received by healthcare professionals

Similarly to healthcare professional students, most healthcare professionals were not educated on medicine disposal and had not received training or education according to three studies.^25^^,^^28^^,^^34^ Less than 30 % of mixed healthcare professionals (pharmacists, nurses, doctors and dental therapists) in a study in Tshwane city admitted to having undergone formal or informal training regarding medication disposal,^34^ while only 11 % of pharmacists in a study in North India were ever taught drug disposal techniques in pharmacy school.^28^ One study conducted in Malaysia recorded that approximately 56 % of community pharmacists had received education for handling unused and expired medications, with most of this training occurring more than a year before the study.^25^

#### 3.2.4 Healthcare professionals' attitudes towards their responsibility and safe disposal

The attitude of healthcare professionals, mainly pharmacists, towards their responsibility in ensuring safe disposal was mixed. Most practising pharmacist respondents in studies from Malaysia and Saudi Arabia believed that they were responsible for ensuring correct medication disposal among patients.^25^^,^^30^ However, there was disagreement about pharmacist's role in disseminating disposal information to the public, with only 23.8 % of private sector pharmacists and 38.5 % of public sector pharmacists in a study in Trinidad agreeing that this duty falls to them.^19^ This sentiment was reflected in responses from the pharmacy and some nursing students in Riyadh and Kosovo as discussed earlier in this review.^40^^,^^44^ Pharmacists in New Delhi were also the lesser group (66 %) as compared to doctors (95 %) and nurses (94 %) who responded positively to the statement that “everyone” was responsible for the disposal of UEMs,^31^ suggesting that many pharmacists believe this responsibility should fall to specific parties rather than being a universal obligation.

#### 3.2.5 Healthcare professionals' professional disposal practices for workplace pharmaceuticals (including patient returns)

According to five studies, good professional practice of pharmacists in handling expired medications was reported.^19^^,^^28^^,^^30^^,^^33^^,^^35^ Three studies found that over 70 % of pharmacists returned their expired medications to pharmaceutical distributors^19^^,^^28^^,^^30^ while around 30 % of pharmacists in the other two studies did the same.^33^^,^^35^ Notably, 30 % to 45 % of those pharmacists from the United Arab Emirates (UAE) disposed of their medications through contractors^33^ and around 30 % to 35 % used the National Agency for Food and Drug Administration and Control (NAFDAC) bins in Nigeria for their drug waste.^35^

#### 3.2.6 Healthcare professionals' practice of advising others on medication disposal or accepting medication returns from patients

The results of this review show that healthcare professionals are not frequently educating their patients nor accepting unused and expired medications for disposal. According to three studies, less than a third of mixed healthcare professional respondents (including pharmacists, doctors, nurses and dental therapists) had regularly counselled their patients regarding safe disposal,^19^^,^^34^^,^^46^ with around 40 % only intermittently counselling their patients.^34^^,^^46^ Interestingly, 72 % of public sector pharmacists in a study in Trinidad reported advising on medication disposal, as opposed to a fifth of private community pharmacists.^19^ Only 10 % of Malaysian community pharmacists stocked promotional materials purveying safe disposal in their pharmacies.^25^ Furthermore, only 8.0 % of doctors, nurses and pharmacists in a study in New Delhi had received leftover medication from their customers,^31^ with 33 % of pharmacists admitting to having rejected patients' medicine returns before.^32^

### 3.3 KAP differences among healthcare professionals and healthcare professional students

Five studies compared KAP outcomes between different categories within their survey populations.^31^^,^^36^^,^^37^^,^^40^^,^^43^ Three studies involving students examined knowledge and awareness among pharmacy students compared to other healthcare professional students,^37^ nursing students,^40^ and general students.^43^ The study by Akande-Sholabi and colleagues found that pharmacy students in Nigeria had significantly higher knowledge than Medical Laboratory Science students, though not significantly different from Medicine and Surgery, Nursing, and Physiotherapy students.^37^ Conversely, the study by Bashatah and Wajid in Riyadh, Saudi Arabia reported that nursing students had significantly greater awareness of the environmental impact of improper disposal compared to pharmacy students.^40^ However, pharmacy students in a study in Bangladesh showed significantly better knowledge than general (engineering and business administration) students regarding the consequences of improper disposal and awareness of standard disposal methods.^43^

A study in New Delhi, India by Bhayana and colleagues revealed that nurses had better knowledge of recommended disposal methods than doctors, and also practised proper disposal more effectively than pharmacists.^31^ Furthermore, a study by Sarraf and colleagues in Dharan, Nepal indicated that junior residents had superior attitudes and practices towards medication disposal compared to faculty members.^36^

### 3.4 Risk of bias assessment

The quality assessment of the articles included in this review was done using the Appraisal tool for Cross-Sectional Studies (AXIS) and the results are shown in Table 5. All included studies have clear reporting for the items studied in the checklist. The sample size for simple random sampling of one article conducted in North India was not justified,^28^ and one study conducted in southwest Nigeria used census sampling for a small pharmacist population.^35^ The validation or piloting of the survey instrument used in one study conducted in India was not reported.^46^ Precision estimates and statistical significance determination methods could not be identified in five studies.^28^^,^^29^^,^^34^^,^^35^^,^^46^ Two studies did not acknowledge the internal consistency of their results caused by missing data.^39^^,^^46^ The study limitations were not discussed in two articles,^35^^,^^46^ and clarifications for any funding received or conflicts of interest were missing from three articles.^28^^,^^36^^,^^46^

## 4 Discussion

This review examined the level of knowledge, attitude and practice of both global healthcare professionals and healthcare professional students concerning the proper disposal of medications. The KAP levels across articles included in this review were similar from various regions, including Africa,^34^^,^^35^^,^^37^^,^^41^ Middle East,^29^^,^^30^^,^^33^^,^^38^^,^^40^ South Asia,^28^^,^^31^^,^^36^^,^^42^^,^^43^^,^^46^ Southeast Asia,^25^ Caribbean,^19^ Europe,^32^^,^^44^ and the USA,^39^^,^^45^ with no major differences among respondent subgroups. However, the knowledge scores were noticeably lower in cohorts consisting of both general and healthcare professional students compared to those comprised solely of healthcare professional students, indicating a higher level of awareness among the latter group. Despite satisfactory environmental attitudes and awareness, healthcare professionals and healthcare professional students across regions demonstrated common deficiencies in knowledge and practices related to safe medication disposal, highlighting an urgent need to address these gaps to improve medication disposal practices globally.

### 4.1 Knowledge of healthcare professionals and healthcare professional students

The review finds a generally satisfactory understanding among both healthcare professionals and healthcare professional students regarding the environmental and health risks associated with improper disposal of medicines. However, while healthcare professionals and healthcare professional students exhibit a superior awareness of these risks compared to the general public,^47^^,^^48^ there exists mixed knowledge concerning the correct disposal channels. For instance, only around 50 % to 65 % of healthcare professionals and healthcare professional students are aware that unused or expired medications should be returned to pharmacies for proper collection and destruction.^29^^,^^36^^,^^37^ This suggests that, despite the advanced education of healthcare professionals and healthcare professional students, key aspects of medication disposal protocols are not being adequately integrated into their practice or curricula. In comparison, less than 25 % of general students demonstrated an awareness of drug take-back programmes,^45^ highlighting a broader public knowledge deficit on this issue. Moreover, there's a surprising lack of awareness among pharmacists regarding the recommended method of incineration for pharmaceutical waste disposal.^34^ A shocking 60 % of pharmacists also thought of medicine disposal into garbage as correct^19^^,^^28^ while 66 % lacked knowledge that contractors and distributors employed incineration for the destruction and inactivation of pharmaceutical waste.^33^These findings suggest that, despite their specialised role in medication management, pharmacists may not be fully informed about essential waste disposal protocols.

The observed mixed knowledge among pharmacists regarding medication disposal can be attributed to a significant gap in education and training on safe medication disposal practices within academic curricula. Studies indicate that less than half of healthcare professionals and healthcare professional students receive formal training on safe medication disposal practices during their academic careers.^25^^,^^28^^,^^34^^,^^37^, ^38^, ^39^^,^^41^ Pharmacists, in particular, often receive limited instruction on this topic both during their undergraduate studies and Continuing Education programmes.^49^ Medication disposal and its importance are frequently overshadowed by a stronger focus on clinical knowledge and consultation skills. This gap in pharmacists' knowledge hinders their ability to actively counsel patients on safe medication disposal,^17^^,^^25^ thereby restricting the development of public awareness regarding the importance of proper HPW disposal and the critical role pharmacies play in environmental stewardship. Given the increasing threat of environmental damage from pharmaceutical waste, it is crucial to integrate medication disposal education into the curricula of healthcare professionals. Continuing Professional Development (CPD) through its learning activities promotes a positive attitude towards life-long learning, and if incorporated with topics focused on safe medication disposal will help healthcare professionals to learn more about this important issue. This, in turn, empowers them to play a vital role in preserving and protecting the environment.^28^ A study has shown that brief educational interventions, such as pharmacy newsletters, can significantly improve pharmacists' knowledge and attitudes towards proper disposal practices.^49^ Therefore, strengthening conventional healthcare education by incorporating comprehensive training on medication disposal and emphasizing the role of healthcare professionals in advocating for proper disposal practices is urgently needed.

### 4.2 Attitude, perceived responsibility and involvement in ensuring safe medication disposal

Many healthcare professionals and healthcare professional students, especially pharmacists, acknowledge their responsibility for ensuring safe medication disposal. This positive attitude likely stems from their environmental awareness and concerns about the impact of improper medication disposal on the environment and water quality.^19^ However, despite recognising their responsibility, they do not always perceive themselves as responsible for educating patients on this matter. Instead, the Ministry of Health is often seen as the primary entity responsible for public education, rather than individual healthcare professionals.^40^^,^^44^ Only 28.4 % of pharmacists believe that providing medication disposal education is part of their job description.^19^ Furthermore, there's a discrepancy between the perceived responsibility and the actual provision of education to patients, with only a third of healthcare professionals consistently providing such education.^19^^,^^34^^,^^46^ This lack of advice received is a significant factor contributing to the public's poor knowledge and subsequent inadequate medication disposal practices.^50^^,^^51^ Research shows that patient are more likely to dispose of medications properly when advised by their healthcare professionals.^18^ Thus, it is crucial to bridge this gap by encouraging healthcare professionals to educate patients on safe medication disposal practices.

Despite conflicting opinions on their obligation to educate the public on proper medication disposal, healthcare professionals, particularly pharmacists, widely support implementing medication take-back programmes. Many pharmacists view community pharmacies as suitable sites for collection depots, suggesting the placement of secure bins within pharmacies.^30^ However, several practical challenges are hindering the success of these programmes, including high costs, ambiguous regulations, and lack of support from the government and the pharmaceutical industry.^32^ While developed countries such as the USA, members of the 10.13039/501100000780European Union, Australia, and New Zealand have established regulations for pharmaceutical waste management, other nations face significant challenges. In some, such as Malaysia and Hong Kong, there is a lack of enforcement of such regulations, leading to inconsistent practices. There is a lack of legislative frameworks for coordinating household pharmaceutical waste disposal, resulting in frequent oversight or neglect of proper medication disposal protocols.^52^^,^^53^ To alleviate the burden on pharmacies, it is essential to establish a straightforward, unified procedure at the national level, funded by local authorities and the pharmaceutical industry.^32^ Conducting local studies to address the issues faced by pharmacists can aid regional policymakers in making informed decisions, thereby enhancing professional involvement and increasing the effectiveness of national medication take-back programmes.

### 4.3 Professional and personal disposal of medications

While professional disposal practices within healthcare settings are generally satisfactory, inadequate personal disposal practices are observed among healthcare professional students and healthcare professionals outside of their workplaces. Pharmacists generally dealt with their expired medications, which included medications collected from the public, by returning them to distributors or contractors for proper disposal.^19^^,^^28^^,^^30^^,^^33^^,^^35^ Surprisingly, many healthcare professional students, often use common household disposal methods for unused or expired medications, mirroring the disposal behaviours of the general population.^2^^,^^54^ This discrepancy indicates that, despite possessing adequate knowledge and positive attitudes towards proper medication disposal, healthcare professionals often struggle to translate theory into practice. This highlights the need for improved HPW collection infrastructure and medication take-back programmes to facilitate safe medication disposal, such as year-long collection services through community pharmacies and other convenient locations. For instance, a study showed that the percentage of people who recycle is over 25 % higher when recycling facilities are easily accessible, compared to those with limited access, despite both groups demonstrating an equally strong willingness to recycle.^55^ Furthermore, strategies to promote behavioural change are necessary to increase the utilisation rate of convenient and accessible disposal facilities once they are made available. Social-behavioural models such as the Social Cognitive Theory,^56^ Theory of Planned Behaviour^57^ and the Health Belief Model^58^ can be useful in exploring the internal and external factors influencing decisions to practice safe medication disposal. These models can also guide the design of educational interventions to improve knowledge and attitudes towards medication disposal.

### 4.4 Limitations

This study faces several limitations. The broad nature of the KAP survey, with its diverse assessments across different studies, made it challenging to synthesize an overall KAP level. This was overcome by identifying and individually analysing the crucial themes of KAP frequently assessed in the included articles. Secondly, the studies primarily involved pharmacists and pharmacy students, with relatively fewer studies focusing on other healthcare professionals and healthcare professional students. This limits the generalizability of the findings. Despite this, the results provide valuable insights into the current KAP of healthcare professionals, allowing for the development of targeted intervention strategies to address knowledge gaps and improve attitudes and practices regarding safe medication disposal.

## 5 Conclusions

In conclusion, this systematic review sheds light on the knowledge, attitudes, and practices of both global healthcare professionals and healthcare professional students regarding medication disposal. It identified several deficiencies in their understanding and execution of safe medication disposal practices, highlighting stagnation from learner to practitioner.

Critical gaps in knowledge persist, particularly concerning the correct disposal channels and methods. Furthermore, while healthcare professionals demonstrate a positive attitude towards safe medication disposal, there remains a disconnect between perceived responsibility and actual engagement in patient education and advocacy. Additionally, despite the widespread support for medication take-back programmes, practical barriers including high costs, ambiguous regulations, and lack of governmental and stakeholder support hinder their success.

To address these challenges, it is imperative to enhance educational and awareness initiatives to promote proper medication disposal practices. Strengthening healthcare curricula to include comprehensive training on medication disposal, increasing awareness campaigns, and improving infrastructure for medication collection and disposal are crucial steps towards mitigating the environmental and public health risks associated with improper medication disposal.

## Funding

This work was supported by the Ministry of Higher Education (MoHE) Malaysia Fundamental Research Grant Scheme (FRGS) [FRGS/1/2022/SS10/UNIM/02/6].

## CRediT authorship contribution statement

**Sheng Yuan Hiew:** Writing – original draft, Formal analysis, Data curation. **Bee Yean Low:** Writing – review & editing, Supervision, Formal analysis, Data curation, Conceptualization.

## Declaration of competing interest

The authors declare the following financial interests/personal relationships which may be considered as potential competing interests:

Sheng Yuan Hiew reports financial support was provided by Ministry of Higher Education Malaysia. If there are other authors, they declare that they have no known competing financial interests or personal relationships that could have appeared to influence the work reported in this paper.

## Data Availability

The datasets generated during the current study are available from the corresponding author upon reasonable request.

## Appendix A Supplementary data

Supplementary Material 1: PRISMA 2020 Checklist Supplementary material 1Supplementary material 1

Supplementary material 2 Supplementary Material 2: Search Strategy. Supplementary material 2

Supplementary material 3 Supplementary Material 3: PRISMA Flow Diagram. Supplementary material 3
