# Supplementary material for: A systematic review of the knowledge, attitude and practice of healthcare professionals and healthcare professional students towards household pharmaceutical waste disposal
Source: Explor Res Clin Soc Pharm. 2024 Dec 19;17:100556. doi: 10.1016/j.rcsop.2024.100556 (PMC11773479; doi:10.1016/j.rcsop.2024.100556)
Supplement: Supplementary material 2 — Search Strategy. [file mmc2.docx]

Supplementary Material 2

Search Strategy 10 Nov 2023

1. PubMed

Results 3188

Title/Abstract:

(healthcare professional[Title/Abstract] OR healthcare provider[Title/Abstract] OR pharmacist[Title/Abstract]) AND ((unused[Title/Abstract] OR unwanted[Title/Abstract] OR expired[Title/Abstract] OR excess[Title/Abstract] OR leftover[Title/Abstract]) medication[Title/Abstract] OR medicine[Title/Abstract]) OR pharmaceutical waste[Title/Abstract] OR (medicine disposal[Title/Abstract] OR waste perception[Title/Abstract] OR pollution awareness[Title/Abstract] OR environmental knowledge[Title/Abstract] OR disposal attitude[Title/Abstract] OR disposal practice[Title/Abstract])

Filters: 2014-2023, English

1. Scopus

Results 2677

Title/Abstract/Keywords:

( healthcare OR professional OR provider OR pharmacist ) AND ( ( unused OR unwanted OR expired OR excess OR leftover ) OR ( medication OR medicine ) OR pharmaceutical OR waste ) AND ( perception OR pollution AND awareness OR environmental OR knowledge OR disposal AND attitude OR practice )

Filters: 2014-2023, Article, English

1. Web of Science

Results 4516

Abstract:

((((healthcare OR professional OR provider OR pharmacist) AND ((unused OR unwanted OR expired OR excess OR leftover) OR (medication OR medicine) OR pharmaceutical waste)) AND (perception OR pollution awareness OR environmental knowledge OR disposal attitude OR disposal practice)))

Filters: 2014-2023, Article, English
