# Supplementary material for: A systematic review of the knowledge, attitude and practice of healthcare professionals and healthcare professional students towards household pharmaceutical waste disposal
Source: Explor Res Clin Soc Pharm. 2024 Dec 19;17:100556. doi: 10.1016/j.rcsop.2024.100556 (PMC11773479; doi:10.1016/j.rcsop.2024.100556)
Supplement: Supplementary material 3 — PRISMA Flow Diagram. [file mmc3.docx]

**Identification of studies via databases and registers**

Records removed *before screening*:

Duplicate records removed (n = 1128)

Records marked as retracted article (n = 4)

Records identified from*:

Pubmed (n = 3188)

Scopus (n = 2677)

Web of Science (n = 4516)

Total (n = 10,381)

**Identification**

Records excluded**

Pubmed (n =3156)

Scopus (n = 2579)

Web of Science (n = 3468)

Total (n = 9203)

Records screened

Pubmed (n = 3183)

Scopus (n = 2585)

Web of Science (n = 3481)

Total (n = 9249)

Reports not retrieved

(n = 0)

Reports sought for retrieval

Pubmed (n = 27)

Scopus (n = 6)

Web of Science (n = 13)

Total (n = 46)

**Screening**

Reports excluded:

Scope not household pharmaceutical waste (n = 18)

Not research article (n = 2)

Not KAP model (n = 3)

Comparison study (n =1)

Pre-print article (n = 1)

Total (n = 25)

Reports assessed for eligibility

Pubmed (n = 27)

Scopus (n = 6)

Web of Science (n = 13)

Total (n = 46)

Studies included in review

Pubmed (n = 11)

Scopus (n = 2)

Web of Science (n = 8)

Reports of included studies

(n = 21)

**Included**

*Consider, if feasible to do so, reporting the number of records identified from each database or register searched (rather than the total number across all databases/registers).

**If automation tools were used, indicate how many records were excluded by a human and how many were excluded by automation tools.

*From:*  Page MJ, McKenzie JE, Bossuyt PM, Boutron I, Hoffmann TC, Mulrow CD, et al. The PRISMA 2020 statement: an updated guideline for reporting systematic reviews. BMJ 2021;372:n71. doi: 10.1136/bmj.n71

For more information, visit: <http://www.prisma-statement.org/>
